# The Bumble motivations framework- exploring a dating App's uses by emerging adults in India

**DOI:** 10.1016/j.heliyon.2024.e24819

**Published:** 2024-01-20

**Authors:** Devadas Menon

**Affiliations:** Development and Educational Communication Unit, Gujarat- 380056, India

**Keywords:** Bumble, Dating, Emerging adulthood, Motivations, Online dating, Uses and gratifications

## Abstract

Researchers have called for a better understanding of the differing motivations of dating app users. Whereas these have been described for Tinder, Grinder and Hinge in the Anglosphere, no research examples could be found for Indian users of Bumble. In response, this paper's pathfinder study helps close the gap by developing a Bumble motivation scale. Six primary motivations for using Bumble were identified through the exploratory factor analysis: Love, Socialisation, Ease of communication, Distraction, Trendiness, and Sexual experience. Notably, socialisation and love motivations emerged as more significant drivers for Bumble usage than sexual experiences, which contrasted with prior findings on dating apps. Additionally, age and biological sex differences were observed in the motivations reported by the participants. Men were more likely to use Bumble for sexual experiences, while women were more motivated by ease of communication. Furthermore, age was found to positively relate to motivations linked to love, distraction, trendiness, and sexual experiences. Moreover, the motivations of Love, socialisation, and trendiness were found to positively predict the frequency of Bumble usage. Among these motivations, Love, socialisation, and trendiness emerged as significant predictors of offline dating behaviour among the participants. These findings contribute to a better understanding of the factors influencing the adoption and engagement with Bumble among emerging adults in India. The implications of these findings may aid in refining dating app features and marketing strategies targeted at this specific demographic.

## 1 Introduction

Bumble is one of the most popular Location-Based Real-Time Dating (LBRTD) apps that allow users to meet potential romantic partners nearby. Like any other dating application, Bumble is designed in such a way to expand the users’ social, romantic, and sexual relationships outside their existing network of contacts [[1], [2], [3]]. Bumble was founded by Whitney Wolf Herd, one of the co-founders of Tinder. Whitney Wolf left Tinder in protest against sexual harassment and discrimination and went on to start a new dating app called Bumble [4].

The dating app Bumble was launched in 2014 with identical features to Tinder. However, the differentiating factor is that the app allows only women to make the first move and initiate a conversation [5]. Once a match is made on Bumble, a conversation has to be initiated by the respective woman within 24 h, while men have no control over starting any tête-à-tête [6]. If the woman fails to respond within 24 h, the match disappears. The power play on the female side is a deliberate attempt to give women autonomy in the dating app space. Bumble is a self-proclaimed feminist dating app [[7], [8], [9]]. The idea of the founders of the app was to reduce the unequal dynamics that exist on other dating apps. Whitney Wolfe believed that this approach was essential to address the prevalent issue of online harassment and to create a more respectful and equitable online dating environment. By allowing women to initiate conversations, Bumble aimed to empower them and give them greater control over their online dating experiences, reducing the likelihood of experiencing uncomfortable or offensive interactions that can be common on other platforms like Tinder [4]. This approach aligns with Bumble's mission to foster more respectful and safe connections in the online dating world, which sets it apart from many other dating apps. To some degree, the discomfort and fear of being harassed are minimized; otherwise part of the online medium [10].

For all the biological sex tropes that the app is associated with, Bumble is one of the primary competitors of Tinder. Though the app is based in the United States of America, its usage seems high in countries like India [11]. Therefore, recognizing the women-centered slant in dating apps is of special importance to a country like India [11].

In 2020 Bumble in India hit 4 million users and showed a trend of women making moves over 15 million times [12]. The COVID-19 pandemic further accentuated the usage of dating apps in India, particularly Bumble. A recently published report on “Intimacy in pandemic” by Bumble suggested that COVID-19 has significantly altered youngsters’ motivation for using dating applications. The report further suggested that in terms of physical relationships, youngsters in India are more open-minded to exploring and experimenting than the youth in the USA and Western countries. Also, 60 % of the respondents who participated in the survey indicated that they were looking forward to being more sexually active once the COVID-19 restrictions eased [13].

Indeed Bumble has significantly increased its user base in developing countries like India, but there is an unclear picture of its user motivations. Although Bumble has similar interfaces and features, as that of other dating applications there are meaningful differences in how they have marketed themselves and are likely to influence motivations to use these apps [14]. For example, the dating app, Tinder is widely marketed as a dating app for casual sex or hook-ups [[14], [15], [16], [17], [18]]. On the other hand, Bumble celebrates itself as a dating app designed to challenge ‘outdated heterosexual norms [19].’ Also, the specific app feature that allows the “women to make the first move” is the unique selling point of Bumble. Hence, the motivation to use Tinder and Bumble can vary with how they have been marketed.

The rise of dating apps coincides with "emerging adulthood," a phase between adolescence and full adulthood (typically ages 18–29), where young adults explore their identities and transition into mature life. Dating apps can significantly impact how they approach relationships during this period, providing a convenient means to meet potential partners and discover their romantic and sexual preferences. These apps offer a diverse pool of partners, allowing individuals to explore various relationship styles and improve their social and communication skills in a low-pressure environment, which is particularly beneficial for socially anxious or less experienced individuals. However, as emerging adults undergo personal growth and exploration, their relationship goals and expectations may evolve. Therefore, it's crucial for researchers to comprehend how dating apps are shaping the romantic lives of emerging adults and influencing their overall development during this pivotal life stage.

Dating apps like Tinder and Bumble have witnessed substantial user growth in low and middle-income nations like India, yet there remains a gap in research regarding the motivations of users in these regions. While existing studies have primarily centered on Tinder [2,20], Bumble has received limited scholarly attention. Despite both apps sharing similar features and interfaces, their distinct marketing approaches are likely to shape users' motivations for selecting one over the other.

Drawing upon the framework of uses and gratifications theory, this study attempts to identify the various motivations for using Bumble among Indian emerging adults. Additionally, the study looks into the biological sex and age differences among the motivations for using dating apps, highlighting the need to examine whether these factors influence Bumble's motivations. Furthermore, the study aims to investigate how Bumble's motivations relate to the frequency of app usage and whether they predict offline dating outcomes. By addressing these research questions, the study intends to contribute to the understanding of Bumble usage motivations and their implications for Indian emerging adults' dating experiences.

This study contributes to the existing literature in three ways. First, theories of media usage behaviour indicate that understating user motivations can shed light on the potential outcomes of particular media usage behaviours [[21], [22], [23]]. Prior literature on dating application usage motivations has shown that certain user motivations are similar to traditional and new media usage while dating apps exhibit certain unique motivations [15] [14, 18]. Hence current study attempts to unravel the various motivations for using Bumble, a dating app which is specifically designed for females. By exploring these motivations, the study will enhance our understanding of user behaviour within the context of dating apps and contribute valuable insights to the field.

Second, LBRTD applications like Bumble have been suggested to positively and negatively affect emerging adults' social, psychological, romantic, and sexual lives [24,25]. For example, nowadays, dating applications help in potential matchmaking that can lead to long relationships like marriage [[26], [27], [28]]. On the other hand, dating applications can affect one's life in an undesirable way, such as sexual harassment and bullying [[29], [30], [31]] and vulnerability to sexually transmitted diseases [32,33]. Understanding user motivations can paint a clear picture of the positive and negative outcomes of Bumble usage. Also, understating motivations can help take appropriate steps to combat adverse outcomes [14].

Lastly, most of the literature on dating apps is from either the United States or western countries, limiting their external validity. Scholars observed cross-cultural differences influence dating norms, particularly individualism, and collectivism [28,34]. They have argued that individualistic and collectivistic cultures influence people's interpretation of love and intimacy [35]. Research findings have indicated that in societies with a strong emphasis on individualism, such as the USA and Australia, dating apps tend to be predominantly utilized for pursuing casual relationships or engaging in hook-up interactions. Conversely, in Asian countries like Japan, Korea, and China, where more collectivist cultural norms prevail, these platforms are often employed as a means to facilitate potential marriage arrangements [26,36]. Earlier investigations [37,38] have pointed out that the inclusion of samples from a nation known for its cultural diversity can bolster the external validity of the research. In the current study, we have utilized our sample from India, a culturally diverse and collectivistic country, to increase the study's external validity.

In short, this study seeks to contribute to the existing literature by unravelling the motivations driving the usage of the dating app Bumble among Indian emerging adults. By understanding these motivations, the research aims to shed light on the potential outcomes of using Bumble and its implications for individuals' dating experiences. Additionally, the study addresses the need to consider cultural diversity and external validity in dating app research by focusing on India as a representative of a collectivistic society. The utilization of the uses and gratifications theory and a mixed-method approach enhances the study's methodology, offering valuable insights and opportunities for replication by other researchers in the scientific community.

## 2 Theoretical framework and research questions

### 2.1 Motivations to use Bumble

Recent reports on Bumble have shown that most of its users are 18–29 years old [39,40]. The uses and gratifications theory is a suitable framework for exploring emerging adults' motivations for using Bumble. This theory assumes that people use mass media to fulfill certain needs and desires [41]. Considering media users as active and purpose-driven in their media choices and usage, the Uses and Gratifications Theory provides a clear insight into user perceptions, behaviours, and outcomes [42,43]. If the media gratifies the users' needs up to their expectations, the users will continue depending on the same media resulting in recurring usage of the media [41,44,45]). For instance, if a user with a sexual motive uses Bumble to gratify their need and is satisfied with the app will be more likely to continue using Bumble. Prior literature has underscored that social, psychological, and physical gratifications motivate users’ consumption of dating apps [15] [8,14,18,46,47]).

Considerable literature has explored the motivations behind the usage of dating applications. The earliest studies in this regard focused on Grindr, the first-ever mobile-based dating application [48]. found that “killing time’” was the most prominent motivation to use Grindr, followed by “making friends,” “having sex/hook-up,” “finding out dating companion,” and “connecting to the gay community.” With the aid of the uses and gratification theory [47], identified six motivations behind Grindr usage: social inclusion, sex, entertainment, friendship, romantic relationships, and location-based partner searching. Other researchers have also reported identical findings [49,50], suggesting that Grinder gratifies its users’ various social, psychological, and physical needs.

Three important scales emerged from three research groups that gained more attention and were widely replicated to capture the gratifications from dating applications. The first scale was developed by Ref. [18]; popularly known as the Tinder Motive Scale (TMS). The TMS was developed through qualitative and quantitative methodology and has identified 13 motivations for using Tinder, i.e., social approval, relationship-seeking, sexual experience, flirting/social skills, travelling, Ex, Belongingness, socializing, peer pressure, sexual orientation, pass time/entertainment, distraction, and curiosity. The second measure of dating applications’ motives is the Motivation to Use Online Dating Scale (MODS) designed by Ref. [51]. MODS reported five motivations behind the usage of dating applications/Tinder, i.e., romantic relationships, casual sex, making new friends, keeping in touch with existing friends, and fun. The final scale for measuring dating apps usage motivations was the Dating App Motivation Scale (DAMS) by Ref. [52]; which was a reclassified version of the original six Tinder motivations (i.e., love, casual sex, ease of communication, self-worth validation, the thrill of excitement, and trendiness) developed by Ref. [14]. In DAMS, the six Tinder motivations were classified under three goals: relational, interpersonal, and entertainment.

An analysis of the various motivations for using dating applications across the three dating app scales shows considerable overlap. In uses and gratification research, the overlap of gratifications among the same media or between different media is a commonly observed phenomenon [53]. In light of prior literature, the motivations for dating apps can be classified under three broad categories (relational, interpersonal, and entertainment), as suggested by Ref. [14]. Most literature on dating applications identified motivations under these broad categories [54]. However, motivations encapsulated under these three classifications have certain inherent lacunas that limit their generalizability.

Most of the prior literature on dating applications has exclusively focused on Tinder. Given its huge popularity across the globe [39,55,56], it is not surprising that most of the prior researchers have specifically focused on Tinder [14,18,51,[57], [58], [59], [60], [61]]. A recent meta-analysis of 70 studies on dating applications has revealed that 35 % of the studies were Tinder-specific [2]. Bumble is second in terms of its user base in developed countries like the USA and developing countries like India [62]. Despite its popularity, no study has yet probed into its user motivations. Recent studies [2,51] speculate that individuals may have different motivations than Tinder for using other dating applications, and studies on other dating applications are highly warranted. A recent study [54] that compared the motivations behind using three dating applications (i.e.Tinder, Bumble, and Hinge) revealed considerable differences in the motivations to use various dating apps.

Further, Bumble's marketing strategies and features (i.e. first move by women) can also influence user motivation. Since no studies could be found that describe Bumble users' motivations, this study intends to uncover the motivations to use Bumble. We believe that the results of this explorative study will be used to create one of the first measures of Bumble motivations. Thus based on the existing literature on dating applications reviewed above, we will present the following research question (RQ).

RQ1 What are emerging adults' primary motivations for using Bumble?

### 2.2 Demographics and Bumble motivations

Literature on gender socialisation has reported that biological sex can be important in determining individuals' social, psychological, and physical needs [[63], [64], [65], [66], [67]]. Studies have observed biological sex and age differences in users’ social, psychological, and physical motivations for dating applications [2,14,54]. For instance, several prior scholars [63]; [15][14,20,51,52,55,68]; Timmermans & De Caluwé, 2017s) have reported that men are more likely to use dating applications to fulfill their sexual needs than women. Similarly, men exhibited more interest in using Tinder for relationship-seeking, love, flirting, thrill-seeking, and ease of communication [20,52,54]. But in another study [18], found no significant difference between men and women in their entertainment motivations. These inconsistencies in prior literature demand further analysis of the biological sex differences among the motivations to use Bumble. It is also worth noting that Bumble is an exclusive application for women where women make the first move.

Age is another important determinant that influences the motivation to use dating apps. Prior research has proven that individuals’ social, psychological, and sexual needs change as they grow older [[69], [70], [71], [72]]. For example, emerging adults tend to have more serious romantic relations once they grow old [[73], [74], [75], [76]]. Also, the tendency to engage in casual sex and hook-up relationships is common while emerging adults grow up [[77], [78], [79], [80]]. Prior studies on dating apps [2,14,52,68] also found significant correlations between age and various motivations to use dating apps. For example [14], found that age positively correlates with motivations like love, casual sex, and ease of communication. Hence it is plausible that the age of Bumble users may have relationships with their usage motivations.

In quintessence, the second research question RQ2 reads.

RQ2 Do the motivations to use Bumble vary with the age and biological sex of the emerging adults?

### 2.3 Bumble motivations and offline dating

Different motivations to use media can result in different outcomes [[81], [82], [83], [84]]. Studies on dating apps [2,14,52,85,86] have revealed that various social, psychological, and physical motivations can influence their usage behaviours. Prior studies [3,14,52,87,88] have looked into the outcomes of dating applications in terms of physical meetings/dating with partners and engaging in casual sex [49]. found that those users who sought to gratify their physical needs met their dating partners and had casual sex more frequently than those who were not motivated to use dating apps for physical gratification. However, results from collectivistic and Asian countries paint a slightly different picture of the motivations and outcomes of dating app usage [28]. Prior research in China, Japan, and Korea has shown that people use dating apps mainly for relationship-seeking and socialisation motivations [36];[89]. Their offline dating often ends in committed relationships or marriages [26]. Prior research on dating apps is largely biased toward Tinder [2,54]. Tinder is considered a masculine app and is largely used for casual sex and hook-ups [14,18].

On the other hand, Bumble is projected and marketed as a female-friendly dating app [7] where women decide to date. Hence the possible outcomes of Tinder can't be generalised to Bumble. Hence in the current study, we try to delineate how Bumble motivations predict offline dating. To sum up, we pose our third and fourth research questions.

RQ3 How do Bumble motivations related to the frequency of usage?

RQ4 How do Bumble motivations predict offline dating?

To address these research questions, we followed the commonly used dual method [90];[43] in uses and gratification research by conducting a focus group interview followed by a survey method. The details are reported below.

## 3 Methods

### 3.1 Focus group summary

Focus groups are commonly used to investigate gratifications due to their methodological advantages. Utilizing an open-ended interview approach with a limited number of participants who possess diverse levels of experience, focus groups offer an effective means of exploring a broad spectrum of ideas that might be challenging to access through alternative research methods [91]. This approach is especially valuable when the objective of the study is to uncover participants' interpretations and modes of comprehension [92]; [80]

Given the constraints imposed by the pandemic, we adapted our research approach and conducted two online focus groups using Zoom. A total of 12 participants took part in these virtual sessions. Based on the availability of participants,7 were assigned to the ﬁrst session and 5 were in the second. The recruitment of participants for our study was carried out through prominent social media platforms like Facebook, Instagram, WhatsApp and Snapchat, as well as through the personal social network of the primary author. Utilizing these channels, we were able to effectively reach and engage individuals who expressed an interest in participating in our research. 7 out of the 12 participants were females and their ages varied from 19 to 26 (M = 23.11, SD = 2.11).

We developed a focus group protocol based on the uses and gratifications framework. By focusing on key concepts such as gratiﬁcation sought, frequency of use, and unintended outcomes, we seek to capture the whole user experience of the participants with Bumble to better identify sources of gratiﬁcations. The focus group discussions were probed using the grounded theory approach (Glaser et al., 1968; Martin & Turner, 1986; Holtzblatt & Beyer, 1998) to tap all the possible motives behind Bumble usage. The obtained themes were further analysed through the lens of prior uses and gratification literature on dating applications. Responses that reflected similar motives were combined, resulting in the re-wording of some of the items. The final item pool consisted of 49 items (see **Anexure-I**). The result revealed that most motivations were similar to previous uses and gratification research on Tinder, such as love, socialisation, ease of communication, distraction, trendiness, sexual experience, the thrill of experience, self-worth validation, entertainment and relationship seeking. As suggested by past researchers [90]; [43], each of the 49 motivations from the 10 themes from the focus groups was then converted into a questionnaire for final data collection.

### 3.2 Sample and procedure

Since the exact statistics of Bumble users in India are unknown, participants were recruited through a convenient snowball sampling method. The snowball sampling method is the easiest and most parsimonious method of data collection from a large and unknown population [93,94]. In researching Bumble usage among Indian emerging adults, where sensitivity and access challenges may arise [95,96] the strategy involves employing individuals' social networks to reach a population considered "hard-to-reach." The term "hard-to-reach" denotes groups difficult for researchers to access due to privacy concerns or cultural factors. Leveraging social connections helps overcome these challenges by tapping into existing relationships and social groups, potentially facilitating access to individuals who may be hesitant or challenging to reach through conventional research methods. Studies of sensitive subjects have employed individuals' social networks to access ‘‘hard-to-reach’’ and ‘‘sensitive’’ populations [97].

All procedures performed in this research involving human participants were in accordance with the 1975 Helsinki declaration. The study is approved by the IRB. The respondents were informed of the objective and purpose of the study. Before conducting the interviews, the respondents’ verbal consent was obtained. The survey instrument was designed in the English language. The potential samples were recruited by posting flyers containing the survey links on various social media platforms like Facebook, Instagram, and WhatsApp. The first page of the questionnaire explained the study objectives and anticipatory benefits. Those respondents who gave their consent by clicking “YES, I wish to proceed” were continued to the next page. Participants were also assured that the survey was anonymous and they could quit anytime. Participation in the survey was voluntary, and participants did not receive any monetary benefits or gifts. The survey was conducted between February to July 2022. The respondents who completed the surveys likely to be from the more affluent strata of Indian society in terms of their mobile phone ownership, daily internet access, English-speaking and affordances to go on regular dates.

Emerging adulthood, a term coined by psychologist [98,99]; refers to a distinct developmental stage experienced by individuals between the ages of 18 and 29. This period is characterized by significant changes and exploration in various aspects of life. One important aspect of exploration for emerging adults is dating and romantic relationships. Unlike individuals in their thirties, emerging adults often have not yet established stable long-term partnerships and instead engage in a series of intimate relationships [98]. In the modern era, dating apps have become increasingly popular among emerging adults as a means to date and initiate romantic connections. A total of 298 (59.7 % males; M_Age_ = 22.23; SD_Age_ = 2.71; Age _Range_ = 18–29) Indian emerging adults, who have been users of Bumble for the last six months, took part in the survey.

The sample size used in the study may be considered small, but the findings can still be meaningful and can be extrapolated under certain conditions. The sample's adequacy relies on its representativeness, context of the study, effect size, and statistical significance [100,101]. Conducting an exploratory factor analysis and ensuring methodological rigour can strengthen the validity of the results [102]. It is essential to transparently report the limitations of the study and acknowledge the potential constraints. While a larger sample would be preferable, the study can offer valuable insights, and replication studies can further confirm and extend the findings.

### 3.3 Measures

3*.2.1. Bumble usage*: Participants indicated their age (in years) and biological sex. Bumble usage of participants was measured using the Tinder usage scale [14]. Participants who were users of Bumble for the last six months were considered for the study. Respondents indicated how often they used the application on a five-point scale ranging from 1 = once or twice in the last six months; 2 = monthly once; 3 = 1–3 times a week; 4 = 4–5 times a week, and 5 = every day. The Bumble usage was normally distributed (Skewness = 0.19; Kurtosis = −0. 84).

#### 3.3.1 Outcome of Bumble usage

The outcome of Bumble usage was measured using the method of [14]. In this method, participants indicated whether they had met a Bumble match for offline dating. The respondents indicated whether they had offline dating or not as a dichotomous variable (1 = Yes; 0 = No).

#### 3.3.2 Bumble motivations

The Bumble motivation scale was developed in a three-step procedure [14] in line with prior studies on dating applications. First, an item pool was created based on the extensive review of prior uses and gratifications literature [14,18,51,52] related to dating apps. The items with similar meanings in the prior literature were used only once. Thus the final item pool consisted of 49 items that represented the various physical, social, and socio-psychological motivations for using dating apps. For each item, the participants were asked to express their agreement on a five-point scale ranging from 1 (*strongly disagree*) to 5 (*strongly agree*). Second, the item pool was reviewed by four (2 male; 2 female) current Bumble users and four (2 male; 2 female) former Bumble users who were not involved in the selection of items to ensure that the items have clarity. Based on the feedback of these independent reviewers, the items were refined. In the third step, exploratory factor analysis was conducted to locate the motivations behind using Bumble.

## 4 Results

### 4.1 Descriptive statistics of Bumble usage and Bumble offline encounters

The frequency of Bumble usage of current and former Bumble users indicated that 61.7 % of the users log on to the app daily. Around 11.7 % used the app once or twice. 10.7 % used the app 4–5 times a week, and 8.7 % used it monthly. Analysis of offline Bumble encounters indicated that more than half of the participants were involved in real-life dating. Men reported higher real-life Bumble dating than women (Refer to Table 1).

**Table 1:** *Offline encounters with Bumble matches* (N = 298).

	Have you been on a Bumble date?	
	Yes	No
Women	46	63
Men	126	63
All	172	126

### 4.2 Bumble usage motivations

A Principal component analysis with Varimax rotation was performed to identify the emerging adults' Bumble usage motivations. A six-factor solution is accepted based on numerous standard criteria such as scree plot analysis, eigenvalues (>1), and interpretability [103]. The factor analysis explained 69.2 % of the variance. All six motivations fulfilled the Kaiser criterion, i.e. eigenvalue>1.0. The items in each interpretable factor, corresponding factor loadings, eigenvalues, percentage of variance explained by each factor, and reliability (Cronbach's alpha) were presented in Table 2.

**Table 2:** Exploratory factor analysis results (N = 298) Bumble.

Motives I use/d Bumble (because) ….	Mean	S. D	Loading	Eigen value	Variance	Alpha
**Love** (Mean = 3.16; SD = 1.33)						
To find someone to be with	2.54	1.57	.794			
To seek out someone to date	3.90	1.10	.761			
To find someone for a serious relationship	2.79	1.38	.755			
To fall in love	2.49	1.41	.743	11.47	30.18	.90
To find a steady relationship	3.76	1.03	.729			
To build an emotional connection with someone	3.09	1.45	.727			
To find a potential romantic partner	3.56	1.42	.700			
**Socialisation** (Mean = 3.15; SD = 1.45)						
To meet new people	3.21	1.47	.919			
To make new friends	3.08	1.46	.854			
To broaden my social networks	3.29	1.47	.809	4.03	10.62	.93
To talk to people, I don't know personally	3.06	1.43	.773			
To get attention	3.06	1.36	.770			
To improve my self-esteem	3.27	1.52	.678			
**Ease of communication** (Mean = 2.52; SD = 1.17)						
Online is easier to open up	2.32	1.36	.837			
Online less shy than offline	2.37	1.39	.836			
People online judge me less	3.97	1.02	.703	3.51	9.25	.83
It is easy to find a match on dating apps	1.44	.916	.670			
**Distraction** (Mean = 2.83; SD = 1.55)						
To distract me from being reminded about my broken relationship	3.12	1.52	.803			
To get out of my otherwise sad/disturbed state	2.79	1.64	.797	3.09	8.15	.89
To feel less lonely	2.68	1.64	.750			
To think less about ex	2.79	1.42	.726			
**Trendiness** (Mean = 2.78; SD = 1.40)						
It is trendy	2.79	1.23	.743			
It is new	2.47	1.46	.634	2.36	6.23	.85
It is cool	2.86	1.58	.618			
Everyone is using it	2.89	1.34	.606			
**Sexual experience** (Mean = 2.66; SD = 1.09)						
To exchange sexy pictures	2.67	1.03	.780			
To meet singles with similar sexual orientation	2.56	1.24	.664	1.80	4.75	.78
To sext	2.78	1.02	.539			

Note: 69.20 % of the total variance explained.

The first motivation for Bumble usage was love, which reflected participants' socio-psychological need to fall in love with someone and build an emotional bond to have a serious relationship. The socialisation motivation was about users' intention to use Bumble to make new connections and broaden their social networks. The ease of communication motivation reflected the users' easiness and comfortability in making an online relationship. The distraction motivation referred to users' intention to use Bumble to forget their past relationships or make them less lonely. The trendiness motivation reflected that some emerging adults find Bumble a cool and trendy app. Finally, the sexual experience motivation sheds light on users’ dependency on Bumble to have online and offline physical and sexual relationships. The intercorrelation between the six motivations is presented in Table 3.

**Table 3:** *Zero-order correlation between Bumble Motivations, Bumble Usage, and Age among Bumble users (N=298)* Zero-order correlation matrix Bumble.

	1.	2.	3.	4.	5.	6.	7.	8.	9.
1. Love	–	.43***	.12*	.47***	.41***	.16**	.24***	.29**	−.13*
2. Socialisation		–	.19**	.41***	.48***	.19**	.34***	.28***	−.06
3. Ease of communication			–	.16**	.26***	−.20***	−.22*	.19**	.08
4. Distraction				–	.56***	.02	−.03	.10	−.15**
5. Trendiness					–	−.09	.20***	.21***	−.27***
6. Sexual experience						–	.04	.12	−.15**
7. Frequency of use							–	.07	−.06
8. Bumble dates (No = 0, Yes = 1)								–	−.26***
9. Age									–

***P < 0.001 **P < 0.01 * P < 0.05.

### 4.3 Biological sex and age differences in Bumble usage

Zero-order correlation, also known as Pearson's correlation coefficient, is conducted to examine the strength and direction of the relationship between two variables. In this case, zero-order correlations were performed to assess the association between age and various motivations for using Bumble. By calculating the correlation coefficient, researchers can determine whether there is a significant linear relationship between age and each motivation and whether the relationship is positive or negative. In the current research, Zero-order correlations showed a significant negative association of age with the motivations of love, distraction, trendiness and sexual experience. That means younger users tend to seek love, distraction, trendiness, and sexual experience gratifications from the usage of Bumble. A MANOVA test (Multivariate Analysis of Variance) was conducted to examine the biological sex differences in Bumble usage motivations among the users. The aim was to determine if there were significant variations between men and women in their motivations for using the dating app. By analyzing multiple motivational factors simultaneously, the MANOVA test provides a comprehensive understanding of the biological sex differences in Bumble usage motivations. MANOVA test (See Table 4) showed a significant biological sex difference for the Bumble usage motivations, i.e., ease of communication and sexual experience, Wilks’λ = 0.53, F (6, 291) = 44.40, p < 0.001, η^2^ = 0.478. Men reported higher sexual experience than women (p < 0.001). On the other hand, compared to men, women reported Bumble being more useful for ease of communication (p < 0.001).

**Table 4:** Mean differences (MANOVA) in Bumble Motivations by biological sex among the users of Bumble (N = 298).

Motive	Biological sex	
	Women	Men
Love	3.05 (0.95)	3.11 (1.14)
Socialisation	3.10 (1.16)	3.19 (1.31)
Ease of communication	3.25 (0.98)	2.10 (0.66) ***
Distraction	3.02 (1.43)	2.74 (1.32)
Trendiness	2.83 (1.10)	3.05 (1.20)
Sexual experience	2.47 (1.01)	2.78 (0.98) ***

***P < 0.001.

### 4.4 Relationship between Bumble motivations and frequency of usage

Zero-order correlations indicated that the frequency of Bumble usage is positively related to love, socialisation, and trendiness motivations. That means emerging adults find Bumble trendy and use it to socialise and find a potential romantic partner.

A hierarchical regression analysis was conducted to investigate the relationship between Bumble usage and motivations in detail. Hierarchical regression analysis allows researchers to examine the unique contribution of each independent variable in explaining the variance in the dependent variable. The use of hierarchical regression analysis in this context suggests that the researchers wanted to investigate the relationship between Bumble usage and motivations while controlling for the potential influence of other variables. By analyzing hierarchically, this study examines the incremental variance explained by each set of variables and determines their relative importance in predicting the outcome. In hierarchical regression, the independent variables are entered into the model in sequential blocks or steps based on theoretical or logical considerations. Typically, demographic variables or control variables are entered in the first step to account for their effects. Then, motivations for Bumble usage were added in subsequent steps to assess their additional contribution to explaining the variance in Bumble usage. The results are presented in Table 5.

**Table 5:** Hierarchical Regression analysis predicting the frequency of Bumble usage.

	Usage (β)
*Controls*	
Age	.009
Bilogical Sex (Male = 1)	.254***
*Predictors*	
R^2^	.063
Love	.152**
Socialisation	.144**
Ease of Communication	−.279
Distraction	−.169
Trendiness	.379***
Sexual experience	.020
R^2^	.183
Total Adjusted ΔR^2^	.160

***P < 0.001 **P < 0.01 * P < 0.05.

The result showed that biological sex is a significant positive predictor of the frequency of Bumble usage (P < 0.001), indicating that men exhibited a higher frequency of Bumble usage (R^2^ = 0.063). Amongst the motivations, love (P < 0.01), socialisation (P < 0.01), and trendiness (P < 0.001) significantly predicted Bumble usage (R^2^ = 0.183).

### 4.5 Relationship between Bumble motivations and Bumble outcomes

The zero-order correlation results (see Table 3) showed that love, socialisation, ease of communication, and trendiness positively related to offline dating. A logistic regression analysis was performed to examine the relationship between Bumble motivations and offline dating experiences. Logistic regression analysis is used to examine the relationship between a binary dependent variable (in this case, offline dating experiences) and one or more independent variables (Bumble motivations) while controlling for the influence of other variables. It is particularly useful when the dependent variable is categorical or dichotomous, and the researcher wants to predict the probability of an event occurring. In this context, logistic regression analysis is conducted to investigate the relationship between Bumble motivations and offline dating experiences. By using logistic regression, it is examined how the different motivations for using Bumble (love, socialisation, ease of communication, and trendiness) relate to the likelihood of having offline dating experiences. Age and biological sex were used as control variables. The result is presented in Table 6.

**Table 6:** Logistic regression predicting Bumble dates.

	B	SE *β*	Wald's X^2^(1)	*P*	*e*^*β*^
*Controls*					
Age	−.234	.059	15.88	.000	0.79
Biological Sex	.738	.403	3.34	.067	2.09
*Predictors*					
Love	.454	.166	7.49	.006	0.64
Socialisation	.406	.148	7.52	.006	0.67
Ease of communication	−.172	.194	0.78	.376	0.84
Distraction	−.520	.136	14.73	.050	0.60
Trendiness	.483	.174	7.67	.006	1.62
Sexual experience	−.157	.167	0.89	.348	0.85

The logistic regression result showed that age negatively predicts offline dating. This means that the younger the user, the more they are inclined toward offline Bumble dating. Among the Bumble motivations, love, socialisation, and trendiness have emerged as significant predictors of offline dating.

## 5 Discussion

Recently, LBRTD applications have become a trend in the mobile dating applications landscape, with Bumble being one of the dominant leaders targeting female mobile daters. The current study advances the literature on LBRTD applications, with a specific focus on Bumble and its motivations among emerging adults. The study is theoretically grounded in the Uses and Gratifications (U&G) theory, which provides a framework for understanding users' motivations and how they interact with technology. By integrating the U&G theory, this study offers a more comprehensive exploration of the motivations driving Bumble usage among emerging adults.

To ensure methodological rigour, a mixed-method approach combining surveys and factor analysis was employed. This approach allowed us to systematically explore and categorize motivations. The survey data was collected from a diverse sample of emerging adults, enhancing the generalizability of findings.

Regarding the first research question, i.e. the emerging adults' primary motivations for using Bumble, the current study located six Bumble motivations: love, socialisation, ease of communication, distraction, trendiness, and sexual experience. Among the six motivations, love, socialisation, and distraction were the prominent ones that drove Bumble dating. Prior studies [2,14,18,52] demonstrated emerging adults’ quest for love and romantic relationships make them expand their dating pool outside their pre-existing networks through mobile dating applications. Similarly, our study echoes prior studies [104,105] that identified mobile dating application users as sociable. This finding implies that for people looking to expand their social capital [106], mobile dating applications can be considered another way of social networking.

By identifying ease of communication as one of the motivations behind Bumble usage, the current study upheld the viewpoint [51] that emerging adults increasingly depend on mobile dating applications for interpersonal goal motivations. Many of the prior studies on Tinder [14,18,52] observed ease of communication as a prominent interpersonal goal for online dating.[14] suggested that mobile dating application users feel more comfortable in online communication where there is a lesser chance of meeting the matches. Break-ups are an inevitable part of dating, whether online or offline. Prior studies [18,107,108] have shown that emerging adults often distract themselves from getting over breakups by using dating applications. In line with these prior studies on mobile dating applications, the current study identified distraction as one of the motivations for emerging adults gratifying from Bumble usage [109]. suggested that online dating has changed over the years from a socially stigmatised practice to a new trend and a preferred way of meeting potential dating partners. Corresponding to this, the last two psychosocial motivations behind using Bumble identified in this study are trendiness and sexual experience. This finding corresponds to the prior uses and gratification studies [18,52] on Tinder.

The current study sheds some light on the influence of culture on dating practices. Dominant motivations identified for using Bumble in the current study are love and socialisation. This finding contradicts the existing scholarships (see Refs. [14,18,52] for mobile dating applications, where the major motivations behind using dating applications were identified as casual sex or a one-night stand. Interestingly, psychosocial motivations like casual sex and one-night stands did not emerge as significant themes in the factor analysis. This finding underscores the influence of cultural differences [28,34] on online dating behaviours. According to Ref. [110]; Indians exhibit a collectivistic culture compared to the United States and western countries’ individualistic cultures. That means Indian users are more likely to be engaged in social media platforms for socialisation and relationship building [45,110]. Along similar lines [35], argued that collectivistic and individualistic values influence the way people interpret love and intimacy and that, in turn, affects their online dating behaviours. Comparative studies on online dating applications [28,36,89] between North American and Asian countries have shown that American daters preferred casual and open relationships compared to their Asian daters, who preferred romantic and committed relationships. It is also observed that online intimacy and romantic relationships in Asian countries often lead to marriages [26]. The difference in Bumble motivations in the current study can also be attributed to rapid progress in the architecture of mobile dating applications where some of the existing gratifications may diminish, reinforce, or an entirely new gratification may emerge [53].

The second research question of the study pertains to the age and biological sex differences in the reported motivations to use Bumble. The findings indicate that younger users seek love, distraction, trendiness, and sexual experience gratifications from using Bumble. In a prior scholarship [111], observed that people increasingly seek out both psychosocial and physical motivations in their relationships during their early adulthood stages. This study also aligns with [61], in which the researchers observed that youngsters identify popular motivations for using Tinder, including entertainment, curiosity, casual sex, and ego-boosting, with 'casual sex' ranking higher in the Indian context.

Significant biological sex differences were also observed in Bumble usage motivations, where men reported higher sexual experience than women. This result is consistent with earlier studies on dating applications [2,14,18,20] that reported that men are more likely to use dating apps for casual sex or hook-ups. The current study reported that women find Bumble more useful for ease of communication. This finding contradicts the prior scholarships [14,52] that have observed men were more likely than women to report ease of communication as a motivation for using Tinder. This difference in motivations for Bumble can be attributed to its design architecture, where only women can make the first move. From the results of the current study, one needs to assume that the ‘women-first’ design architecture of Bumble allows women to screen and filter all their requests and accept the best ones so that interpersonal communication with the prospective match can be smooth.

The biological sex differences observed in Bumble usage motivations, particularly regarding ease of communication and sexual experience, can also be illuminated by insights from the sociology of online dating and evolutionary psychology [112]. extensively explores the connection between evolutionary psychology and human behaviour, particularly in the realm of dating and mating. Buss's research suggests that certain dating and relationship behaviours can be traced back to our evolutionary past. For instance, the higher reported motivation among men for sexual experiences on Bumble aligns with the idea that their drive for mating and competition, as proposed by Ref. [112]; may influence these preferences. This perspective implies that men might view online dating platforms as avenues for pursuing short-term, casual encounters, thereby explaining their heightened focus on sexual experiences as a motivation. Further, insights into sex differences in dating preferences, such as the importance of employment status, education, and religious beliefs, offer valuable context for understanding the motivational disparities between women and men in the study [113]. According to Ref. [113]; men's focus on employment status and women's varied preferences related to education, family aspirations, and religious beliefs underscore the multifaceted nature of dating motivations. These findings emphasize the complex interplay of cultural and personal factors, suggesting that the motivations for using Bumble may be shaped by a diverse range of considerations, including communication, shared values, and economic and societal expectations.

The third research question of the study was to find out the relationship between Bumble motivations and the frequency of Bumble usage. In line with the prior scholarship [114], biological sex emerged as a significant positive predictor of Bumble usage, indicating that men exhibited a higher frequency of Bumble usage. This result supports the reports [115,116] on the usage of Bumble in India in which men were the largest users of Bumble and exhibited a higher frequency of usage. Furthermore, the higher frequency of Bumble usage among men may be influenced by societal norms and expectations [117,118]. Men, on average, might feel more comfortable being proactive in their search for potential partners, leading them to use dating apps more frequently. On a similar line [64], suggested that men prefer online communication as an easier way to meet new people and potential romantic partners than women. Amongst the motivations, love, socialisation, and trendiness significantly predicted Bumble usage. Hence, the current study supports the argument [114];[119] that dating apps are used more for entertainment purposes than sex or dating. [14]; based on their study on Tinder, suggested that some people are more interested in using dating apps to explore their features than their functionality.

Finally, the current research delineated some interesting insights from the relationship between demographics, Bumble motivations, and physical dating. The result showed that age negatively predicted offline dating, indicating that the younger the user, the more they are inclined toward offline Bumble dating [73]. stated that emerging adulthood is a distinct developmental period in an individual life cycle characterised by change and exploration. One of the primary ways through which emerging adults can experience change and exploration is by using online dating apps. [2] suggested that youngsters increasingly depend on dating apps to pursue social, sexual, and romantic connections. Hence, given the ubiquity of smartphone usage, it is logical that the younger the user, the more inclined to invite their prospective matches for a face-to-face meeting [114]. Among the Bumble motivations, love, socialisation, and trendiness have emerged as significant predictors of physical dating. This finding corresponds to the previous studies on Tinder [14,52,119], in which many users tend to use the dating app for entertainment goals and are in line with the prior uses and gratifications studies [120,121] on social networking. The establishment and continuance of romantic relationships online are very common nowadays, and these relationships are similar in meaning, intimacy, and stability compared to offline relationships [122]. In the case of mobile dating applications, relationships developed online can lead to subsequent offline dating that continues to develop over a while into longer romantic partnerships and marriages [26,36,123].

This study offers a valuable methodology for the scientific community by advancing the literature on LBRTD applications, specifically focusing on Bumble, a popular mobile dating app targeting female users. It is innovative because it addresses the motivations behind using Bumble among emerging adults, shedding light on the factors driving its popularity among this age group. The methodology employed in this study can be replicated by other researchers interested in investigating motivations behind LBRTD applications, not just limited to Bumble. Researchers can use a mixed-method approach, combining surveys and factor analysis to identify and categorize motivations. Additionally, by considering cultural differences and demographic factors, such as age and biological sex, researchers can gain insights into how these motivations vary across different user groups.

The study's emphasis on age and biological sex differences in Bumble usage motivations also opens up avenues for comparative research across various dating apps and cultures. By replicating this methodology in different contexts, researchers can contribute to a more comprehensive understanding of how LBRTD apps influence dating behaviours and social interactions among emerging adults. This research approach allows for a deeper exploration of users' intentions and preferences, contributing to the development of user-centric design and targeted marketing strategies for dating applications. Overall, the study's methodology offers a valuable template for researching LBRTD applications, making it a significant contribution to the scientific community studying online dating behaviour and social networking trends.

## 6 Theoretical and practical implications

The current study's findings have several important implications: Theoretically, integrating the U&G theory has identified six potential motivations for emerging adults' usage of the relatively new and trendy but hardly studied Bumble dating app. Significant differences were also observed between the motivations of Bumble and its competitor, Tinder. For example, in the current study, socialisation has emerged as one of the most significant motivations for Bumble usage compared to casual sex motivation ([124] for Tinder. This finding supports the argument that technological affordances may cause existing gratifications to diminish or reinforce, or an entirely new gratification may emerge [53]; [43] Further, this study reinforced the influence of culture [34] on dating applications by not locating casual sex as the major motivation for Bumble usage.

The practical implications of the study call for greater awareness of the technological affordances and marketing strategies of dating applications. Identifying the user motivations behind mobile dating applications is important for advertisers of these applications as they can design their user profiles and marketing strategies. Our findings indicate that people use Bumble for social networking rather than a sexual experience. Hence, to attract more potential users to Bumble, marketers can provide more features and a variety of tools to stimulate social networking among their users further. For example, marketers can facilitate chat rooms or group creation options for their users so that they can form communities for dating. Given our finding that the trendiness motive influences the frequency of Bumble usage and offline dating, the developers should make the apps more cool and trendy to appeal more to potential users. Similarly, since female users find Bumble useful for ease of communication, app designers can think of providing more women-friendly features to attract potential customers.

## 7 Limitations and future research

Our findings should be interpreted in light of several limitations. First, the convenient sampling method, cross-sectional design, and small sample size limit the possibility of generalising our findings. Hence, study results should be interpreted in the cultural context of India. Second, although we have conducted a focus group interview to identify the new sets of motivations, the motivation scale that emerged in the current study is largely identical to existing dating app motivation scales. Self-report bias in the collected data introduces the potential for skewed responses, affecting the reliability of reported motivations. Furthermore, although the study has identified correlations between motivations and usage frequency, establishing causality remains a complex task, potentially complicated by unaccounted-for variables. To enhance the depth of understanding, future research endeavours should focus on building and expanding upon the existing scales, aiming to capture a broader spectrum of motivations underlying Bumble usage. Additionally, the recently developed Bumble scale should undergo cross-validation in an independent sample, with a meticulous examination of both convergent and divergent validity for the six identified motivations. This rigorous validation process will contribute to the robustness and reliability of the motivation scale, ensuring its applicability and accuracy in diverse contexts. Third, the study's exploration of age and biological sex differences should be understood within the context of its specific sample, potentially not fully representative of all emerging adults. Also [125], recommended giving adequate representation to non-binary genders in human-computer interaction (HCI) research. However, this study did not include individuals who identified as transgender, limiting the insights into transgender dating experiences within the LGBTQ + community. This omission underscores the need for future research to explore and address this specific aspect of human-computer interaction.

Further, Bumble's emphasis on women initiating contact may deter men seeking casual encounters (who prefer platforms like Tinder) and could potentially exclude gay men (favouring Grindr) and lesbians as participants, potentially introducing a sample bias. Fourth, the current study measured offline Bumble dating on a dichotomous scale. Also, the study did not include several potential offline dating app behaviours. We suggest that forthcoming researchers incorporate supplementary measures of offline behaviours to gain a comprehensive understanding of Bumble's impact on the lives of emerging adults. Additionally, we recommend that future studies include the quantification of Bumble dates as a key metric, providing insights into the platform's role in facilitating real-world interactions and potentially fostering long-term relationships. This finding can demonstrate whether Bumble facilitates long-term romantic relationships, such as marriages. Fifth, there is a likelihood that the respondents, characterized by mobile phone ownership, daily internet access, English proficiency, affordability for regular dates, and the time and cultural capital for questionnaire completion, represent a more affluent segment of Indian society. This indicates a potential bias towards economically privileged participants. It is imperative to emphasize the need for future research that investigates dating app motivations among less economically affluent strata for a more representative understanding of Indian society's dating dynamics. Additionally, the study could benefit from further exploration of caste and ethnic differences in dating app motivations, recognizing that India's diverse cultural landscape may influence dating preferences differently among various groups.

Sixth, this study did not address the potential presence of deceptive or insincere users on dating apps, including romance scammers, individuals experimenting with false digital identities (e.g., identity play), and instances where individuals misrepresent themselves, such as men posing as women [126]. Additionally, there may be cases of people seeking free meals, a phenomenon sometimes referred to as "foodie calls" or "sneating." These behaviours, if prevalent, could introduce elements of dishonesty and exploitation within the online dating environment. Understanding the extent and impact of such behaviours on the dynamics of dating apps would require further investigation, which future researchers can explore.

Lastly, the present study was set amidst the COVID-19 pandemic. Hence, we believe that the pandemic might have influenced user motivations to some extent. Hence, we recommend another post-pandemic longitudinal study with a more nuanced set of motivations to yield better generalisable results.

## Data availability statement

Data will be made available on request.

## CRediT authorship contribution statement

**Devadas Menon:** Writing – review & editing, Writing – original draft, Validation, Supervision, Software, Resources, Project administration, Methodology, Investigation, Formal analysis, Data curation, Conceptualization.

## Declaration of competing interest

The authors declare that they have no known competing financial interests or personal relationships that could have appeared to influence the work reported in this paper.
