# Corrigendum to “The Bumble motivations framework-exploring a dating App's uses by emerging adults in India” [Heliyon Volume 10, Issue 3, February 2024, Article e24819]

**DOI:** 10.1016/j.heliyon.2025.e43398

**Published:** 2025-05-15

**Authors:** Devadas Menon

**Affiliations:** Development and Educational Communication Unit, Gujarat, 380056, India

In the original published version of this article, the ethics approval details, and the year of data collection were incorrect.

The original manuscript showed the ethics details as below:

All procedures performed in this research involving human participants were in accordance with the 1975 Helsinki declaration. The study is approved by the IRB. The respondents were informed of the objective and purpose of the study. Before conducting the interviews, the respondents’ verbal consent was obtained. The survey instrument was designed in the English language. The potential samples were recruited by posting flyers containing the survey links on various social media platforms like Facebook, Instagram, and WhatsApp. The first page of the questionnaire explained the study objectives and anticipatory benefits. Those respondents who gave their consent by clicking “YES, I wish to proceed” were continued to the next page. Participants were also assured that the survey was anonymous and they could quit anytime. Participation in the survey was voluntary, and participants did not receive any monetary benefits or gifts. The survey was conducted between February to July 2022. The respondents who completed the surveys likely to be from the more affluent strata of Indian society in terms of their mobile phone ownership, daily internet access, English-speaking and affordances to go on regular dates.

The ethics approval committee name, approval number and the year of data collection has been updated.

The corrected version of the ethics can be found below:

All procedures performed in this research involving human participants were in accordance with the 1975 Helsinki declaration. The study is approved by Deenadayalu Janaseva Samithi (R), an independent ethics committee on 6th January 2023, (approval reference: IEC/MHD/0211/23). The respondents were informed of the objective and purpose of the study. Before conducting the interviews, the respondents’ verbal consent was obtained. The survey instrument was designed in the English language. The potential samples were recruited by posting flyers containing the survey links on various social media platforms like Facebook, Instagram, and WhatsApp. The first page of the questionnaire explained the study objectives and anticipatory benefits. Those respondents who gave their consent by clicking “YES, I wish to proceed” were continued to the next page. Participants were also assured that the survey was anonymous and they could quit anytime. Participation in the survey was voluntary, and participants did not receive any monetary benefits or gifts. The survey was conducted between February to July 2023. The respondents who completed the surveys likely to be from the more affluent strata of Indian society in terms of their mobile phone ownership, daily internet access, English-speaking and affordances to go on regular dates.

The authors apologize for the errors.

## Declaration of competing interest

The authors declare that they have no known competing financial interests or personal relationships that could have appeared to influence the work reported in this paper.

