# Supplementary material for: The Bumble motivations framework- exploring a dating App's uses by emerging adults in India
Source: Heliyon. 2024 Jan 20;10(3):e24819. doi: 10.1016/j.heliyon.2024.e24819 (PMC10839884; doi:10.1016/j.heliyon.2024.e24819)
Supplement: Multimedia component 1 [file mmc1.docx]

**Questionnaire**

Dear respondent,

We are conducting a study on the usage of the dating application Bumble. Besides collecting information about your demographic details we also collect a few insights about your motivations behind the usage of Bumble. It will take about 10 Minutes to complete this survey. If you feel any discomfort in filling out the questionnaire, you are free to leave out any question(s), or you are free to quit this survey at any time. We confirm that we are not collecting any personally identifiable details about you and request you not provide any personally identifying information in the questionnaire. We assure you that the responses provided will be kept confidential and not be shared with any third party. We also confirm that your data will be used only for the research purpose mentioned above and will not be used for any other purposes.

If you agree to participate in the survey, kindly click ‘**YES’** to proceed with the survey. If you do not agree, please click ‘**NO’**.

Thank you

(Researcher)

**Part I**

I have read and understood the above study’s objectives and information asked from me and have had the opportunity to ask questions. I understand that my participation is voluntary and that I am free to withdraw at any time, without giving a reason and without cost. I understand that I will be given a copy of this consent form. I voluntarily agree to take part in this study and I wish to proceed:

YES

NO

**Part II**

1. Your gender Male Female
2. What is your age ---------------

**Part III**

**The following sections deal with the motives/ reasons for using Bumble. Kindly provide your reason for using Bumble.**

1. I enjoy browsing on Bumble
2. When I have nobody else to talk
3. When I have nothing better to do
4. Everyone uses Bumble
5. My online Bumble connections understand me better than other people
6. To find out information about other users
7. To look at other user's pictures
8. To look at other user's profile
9. To talk to someone
10. To feel better about myself
11. To find someone to be with
12. To seek out someone to date
13. To find someone for a serious relationship
14. To fall in love
15. To flirt
16. To find a steady relationship
17. To build an emotional connection with someone
18. To find a potential romantic partner
19. To meet new people
20. To make new friends
21. To broaden my social networks
22. To talk to people, I don’t know personally
23. To get attention
24. To improve my self-esteem
25. To gain more self-confidence
26. Online is easier to open up
27. Online less shy than offline
28. People online judge me less
29. It is easy to find a match on dating apps
30. To distract me from being reminded about my broken relationship
31. To get out of my otherwise sad/disturbed state
32. To feel less lonely
33. To think less about ex
34. It is trendy
35. It is exciting
36. It is entertaining
37. It is new
38. It is cool
39. It is fun
40. Everyone is using it
41. To exchange sexy pictures
42. To meet singles with similar sexual orientation
43. To sext
44. To have a one-night stand
45. To relax
46. Out of habit
47. To cheer up myself
48. To see who else uses the application
49. To find someone to have sex with

**Part IV**

**The following section deal with your frequency of Bumble usage and offline dating behaviour in the last six months.**

1. How often are you using/used Bumble in the last six months?

once or twice

monthly once

1-3 times a week

4-5 times a week

Every day

1. Have you ever gone for an Offline Bumble date (physically met the person whom you know through Bumble) in the last six months?

YES

NO

**Thank you for your responses!**
